# Etiology of cirrhosis is associated with risk of hepatic decompensation and hepatocellular carcinoma

**DOI:** 10.1186/s12876-025-04538-y

**Published:** 2025-12-09

**Authors:** Michelle Ng, Olgert Bardhi, Krystal Lai, Eden Koo, Sruthi Yekkaluri, Kevin Bass, Guruveer Bhamra, Pojsakorn Danpanichkul, Lisa Quirk, Ju Dong Yang, Jeremy Louissaint, Thomas A. Kerr, Amit G. Singal

**Affiliations:** 1 Department of Internal Medicine, UT Southwestern Medical Center, Dallas, TX USA; 2 Department of Internal Medicine, Texas Tech University Health Sciences Center, Lubbock, TX USA; 3 Karsh Division of Gastroenterology and Hepatology, Cedars-Sinai Medical Center, Los Angeles, CA USA; 4 Division of Digestive and Liver Diseases, UT Southwestern Medical Center, Dallas, TX USA; 5 Department of Internal Medicine, University of Texas Southwestern, 5959 Harry Hines Blvd, POB 1, Suite 420, Dallas, TX 75390-8887 USA

## Abstract

**Background:**

The impact of the changing epidemiology from viral to non-viral etiologies of cirrhosis on the burden of liver-related complications remains unclear.

**Methods:**

We conducted a retrospective cohort study of adult patients with cirrhosis and an index outpatient visit between January and December 2015 at two U.S. health systems. We excluded patients with a history of hepatocellular carcinoma (HCC) or both prevalent ascites and hepatic encephalopathy. Fine-Gray sub-distribution hazard models were used to characterize time-to-incident hepatic decompensation and incident HCC through 2020, with liver transplantation and death as competing events, and multivariable Fine-Gray regression was used to identify associated factors.

**Results:**

We identified 1029 patients (median age 58 years, 54.9% male, 19.5% non-Hispanic White). Over a median follow-up of 84.7 months, 36.4% developed incident hepatic decompensation (46.7% ascites, 21.1% hepatic encephalopathy, and 32.3% ascites plus hepatic encephalopathy), 14.5% developed HCC, 2.0% underwent transplant, and 23.0% died. The cumulative 1-, 2-, and 3-year incidence of hepatic decompensation were 7.0%, 10.8%, and 16.3% and incidence of HCC were 3.0%, 5.0%, and 6.9%, respectively. Compared to viremic hepatitis C, higher risk of hepatic decompensation was associated with metabolic dysfunction-associated steatotic liver disease (MASLD) (sHR 1.52, 95% CI 0.94 - 2.45) and alcohol-associated cirrhosis (sHR 1.68, 95%CI 1.10 - 2.57), while incident HCC was inversely associated with MASLD (sHR 0.27; 95% CI 0.12–0.59) and alcohol-associated cirrhosis (sHR 0.45; 95% CI 0.23–0.84).

**Conclusion:**

Increasing proportions of non-viral liver disease will likely lead to a greater burden of hepatic decompensation and reduced HCC in contemporary populations.

**Supplementary Information:**

The online version contains supplementary material available at 10.1186/s12876-025-04538-y.

## Introduction

Cirrhosis is the 11th most common cause of death in the world, and it affects approximately 2.2 million adults in the U.S [1, 2]. Cirrhosis occurs due to chronic liver inflammation leading to the replacement of normal liver tissue by regenerative fibrotic nodules [3]. This process causes portal hypertension, which can lead to complications or hepatic decompensations, such as ascites, variceal bleeding, or hepatic encephalopathy [4]. Patients with cirrhosis are at increased risk of hepatocellular carcinoma (HCC), with an annual incidence exceeding 1% across etiologies. HCC is the leading cause of cancer-related death in this population [5, 6].

The epidemiology of cirrhosis is shifting from predominantly viral hepatitis due to chronic hepatitis B virus (HBV) or hepatitis C virus (HCV) to increasing proportions related to metabolic dysfunction-associated steatohepatitis (MASH) and alcohol-associated liver disease (ALD) [7, 8]. Increased HBV vaccination rates have resulted in a decreased burden of HBV-related liver disease and downstream complications, including HCC [9]. Furthermore, HBV and HCV antiviral therapy reduces the risk of progression to cirrhosis and is associated with a reduced risk of HCC [10, 11]. Although viral hepatitis remains the leading cause of cirrhosis worldwide, there has been a growing burden of patients with MASH and ALD, which is driven by the rising prevalence of obesity, diabetes, and alcohol consumption [12–15]. Indeed, metabolic dysfunction-associated steatotic liver disease (MASLD) currently affects a third of the global adult population and is becoming the most common cause of chronic liver disease globally [16–19]. MASLD can progress to MASH, which can then lead to cirrhosis, HCC, and death [20]. The incidence of hepatic decompensation, HCC, and death related to MASH is expected to double by 2030 [21]. Alcohol use has also been increasing, with age-standardized death rates related to ALD projected to increase from 8.2 deaths per 100,000 patient-years in 2019 to 15.2 deaths per 100,000 patient-years by 2040 [22, 23]. Increased alcohol consumption during the COVID-19 pandemic further contributed to the global burden of alcohol misuse and ALD [24, 25].

The impact of this shift in liver disease etiology on the anticipated burden of hepatic decompensation and HCC has not been well studied in contemporary cohorts. We conducted a retrospective cohort study to characterize the natural history of cirrhosis, stratified by liver disease etiology, in a contemporary population of patients in the United States.

## Methods

### Study population

We included a convenience sample of adult patients with cirrhosis who had an index clinic visit between January 2015 and December 2015 at two large urban health systems in the United States: Parkland Health and UT Southwestern Medical Center. Parkland Health is a safety-net health system for underinsured patients in Dallas County, and UT Southwestern Medical Center is a tertiary care referral center in Dallas, Texas.

Patients with cirrhosis were initially identified using a validated set of ICD-9/ICD-10 codes for cirrhosis and its related complications (e.g., ascites, hepatic encephalopathy, portal hypertension, and varices) (Supplemental Table 1) [26]. The index visit was defined as the first clinic visit during the study period after one of the applicable ICD-9/ICD-10 codes was present. The presence of cirrhosis was then confirmed by chart review, with diagnosis defined by consistent liver biopsy, transient elastography, serum fibrosis markers (e.g., FIB-4 or Fibrotest), or imaging showing a cirrhotic-appearing liver with signs of portal hypertension. We excluded patients with both ascites and hepatic encephalopathy at the time of index visit and those with a history of liver cancer. The study was approved by the institutional review board at UT Southwestern Medical Center. A waiver of informed consent was granted given the retrospective nature of the study.

### Data collection

Demographic and clinical characteristics were abstracted from electronic medical records for all patients including age, sex, race and ethnicity, liver disease etiology, and liver disease severity. Race and ethnicity were classified as Non-Hispanic White, Non-Hispanic Black, Hispanic, and other. Liver disease etiology was classified in a hierarchical manner as viremic HCV, post-sustained virologic response (SVR) HCV, HBV, ALD, MASLD, and other. The etiology was cirrhosis was determined by chart review, including the clinical assessment of gastroenterology or hepatology providers. HCV diagnosis was based on the presence of positive antibody, viral load, or history of treatment, and HBV infection was based on the presence of HBsAg and/or anti-HBV treatment. ALD was based on a history of heavy alcohol use, and MASLD was diagnosed by presence of associated metabolic conditions. Liver disease severity was assessed by the Child-Pugh score, with ascites and hepatic encephalopathy classified as none, mild/controlled, and severe/uncontrolled. We recorded dates of all new-onset ascites, hepatic encephalopathy, and HCC, as well as dates of liver transplantation or death. Incident decompensation events were identified through manual chart reviews and recording the earliest date of any new hepatic decompensation.

### Statistical analysis

Our primary outcomes were (1) incident hepatic decompensation, defined as new onset ascites or hepatic encephalopathy and (2) incident HCC, which was defined using American Association for the Study of Liver Diseases (AASLD) criteria, i.e. characteristic imaging per LI-RADS or consistent histology [27, 28]. A key secondary outcome was incident death.

We used Fine-Gray subdistribution hazard models to characterize time to hepatic decompensation and time to HCC, with liver transplantation and death as competing events. For hepatic decompensation models, HCC was also treated as a competing outcome given the risk of hepatic decompensation after HCC treatment. A Fine-Gray subdistribution hazard model was also used to characterize time to death, with liver transplantation treated as a competing risk. Patients were censored at their last follow-up and were accounted for competing events. We used Gray’s test to compare CIF (Cumulative Incidence Function) between etiologic subgroups. Multivariable Fine-Gray subdistribution hazard models were used to identify factors associated with hepatic decompensation, HCC, and death. Age, sex, race, Child-Pugh class, MELD-Na score, BMI, and metabolic syndrome comorbidities were forced into multivariable models given a priori importance. Statistical significance was defined as *p* < 0.05 for all analyses. Statistical analyses were performed using SAS 9.4 (SAS Institute Inc., Cary, NC).

## Results

### Patient characteristics

Of the 1358 patients with cirrhosis seen during the study period, there were 1178 patients with complete data for demographics, liver disease etiology, and survival. We excluded another 3 patients with a history of liver cancer and 146 patients who had both ascites and hepatic encephalopathy. Characteristics of the 1029 eligible patients are detailed in Table 1. The median age of patients was 58 years, and 54.9% were male. The cohort was diverse regarding race and ethnicity (37.9% non-Hispanic Black, 34.4% Hispanic, 19.5% non-Hispanic White) and liver disease etiology (36.3% viremic HCV, 18.1% MASLD, 17.7% ALD, and 13.9% post-SVR HCV). Most patients had compensated cirrhosis at the index visit, with ascites being present in 15.6% and hepatic encephalopathy in 5.0%.

**Table 1:** Patient characteristics

Covariate*	*N* = 1029 (%)
Age (median, IQR)	58 (54–64)
Sex (% male)	565 (54.9%)
Race and ethnicity	
Non-Hispanic White	201 (19.5%)
Non-Hispanic Black	390 (37.9%)
Hispanic	354 (34.4%)
Other	84 (8.2%)
Etiology	
Viremic hepatitis C virus	374 (36.3%)
Hepatitis B virus	20 (1.9%)
Alcohol-associated liver disease	182 (17.7%)
Metabolic dysfunction-associated liver disease	186 (18.1%)
Post-SVR hepatitis C	143 (13.9%)
Other	124 (12.1%)
Presence of diabetes	434 (42.3%)
Presence of dyslipidemia	310 (30.3%)
BMI (median IQR)	30 (25.9–35.1)
Ascites	160 (15.6%)
Hepatic encephalopathy	51 (5.0%)
Bilirubin (median, IQR)	0.7 (0.4–1.2)
Albumin (median, IQR)	3.8 (3.4–4.1)
INR (median, IQR)	1.1 (1–1.2.2)
MELD-Na (median IQR)	9.8 (7.5–17.9)
Child Pugh class	
Child Pugh A	805 (78.2%)
Child Pugh B	224 (21.8%)

*Abbreviations*: *SVR* Sustained virologic response

* Bilirubin was missing in 5.2%, albumin 6.3%, and INR 13.1% of patients. Demographics, etiology, ascites, and hepatic encephalopathy had no missing data

### Incidence of hepatic decompensation and development of HCC

**Fig. 1:**
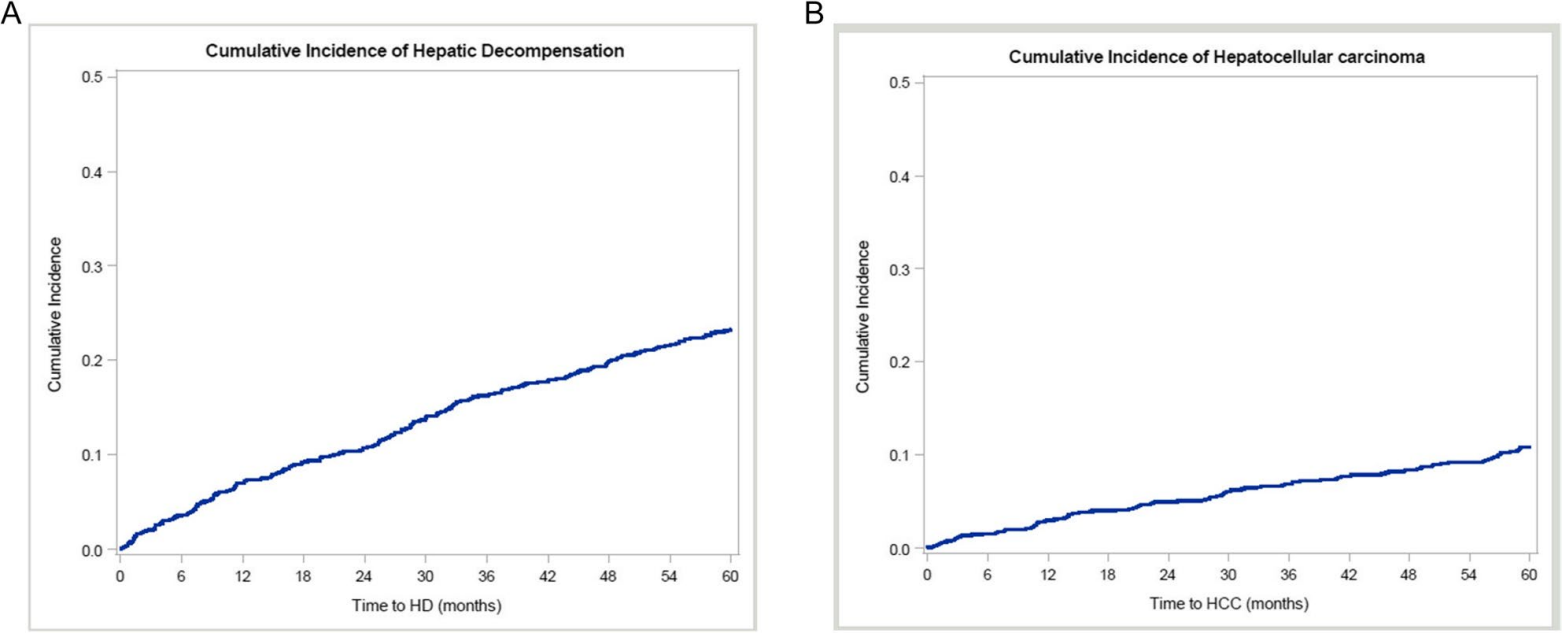
Cumulative incidence of hepatic decompensation and hepatocellular carcinoma. The cumulative 1-, 2-, and 3-year incidence rates of hepatic decompensation (Panel **A**) were 7.0%, 10.8%, and 16.3%, and those of HCC (Panel **B**) were 3.0%, 5.0%, and 6.9%, respectively. Abbreviations: HD, hepatic decompensation; HCC, hepatocellular carcinoma

Patients were followed for a median follow-up of 84.7 months. Incident hepatic decompensation was observed in 375 (36.4%) patients (46.7% new ascites, 21.1% new hepatic encephalopathy, and 32.3% both ascites plus hepatic encephalopathy), and 149 (14.5%) developed HCC. Twenty-one (2.0%) patients underwent LT, and 237 (23.0%) patients died. The cumulative 1-, 2-, and 3-year incidence rates of hepatic decompensation were 7.0%, 10.8%, and 16.3%, and those of HCC were 3.0%, 5.0%, and 6.9%, respectively (Fig. 1A and B). Among the 818 patients with compensated cirrhosis at baseline (no ascites or hepatic encephalopathy), the cumulative 1-, 2-, and 3-year incidence rates of hepatic decompensation were 5.9%, 9.5%, and 14.6%, and those of HCC were 2.6%, 4.4%, and 6.4%, respectively.

### Factors associated with hepatic decompensation

In multivariable analyses adjusted for demographics, liver dysfunction, and metabolic syndrome comorbidities, incident hepatic decompensation was independently associated with liver disease etiology (Table 2). Compared to patients with viremic HCV, those with ALD had a higher risk of hepatic decompensation (sHR 1.68; 95% CI 1.10–2.57). MASLD was also associated with higher decompensation, although this did not reach statistical significance (sHR 1.52; 95% CI 0.94–2.45).

**Fig. 2:**
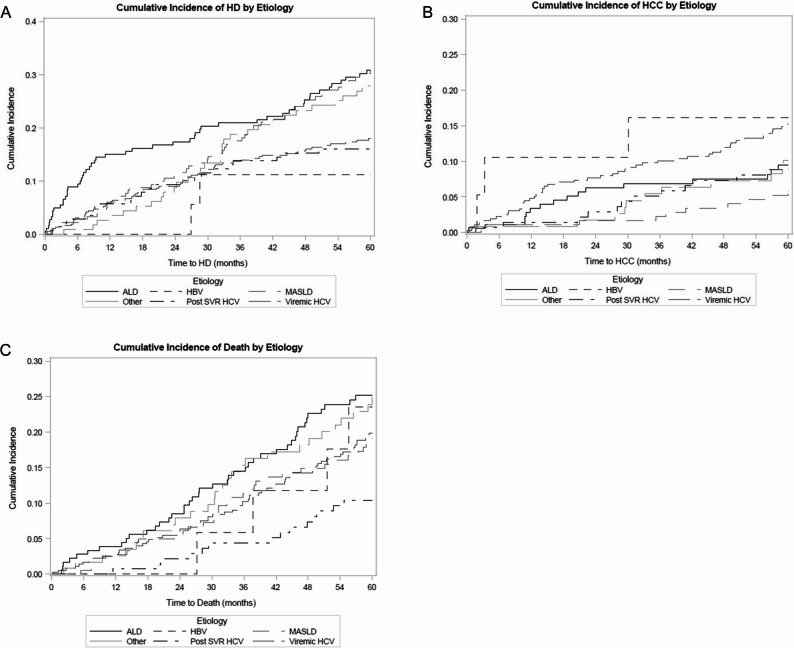
Cumulative incidence of hepatic decompensation, hepatocellular carcinoma, and death, stratified by liver disease etiology. Compared to viremic hepatitis C, patients with non-viral etiologies of liver disease had a significantly higher incidence of hepatic decompensation (Panel **A**) and lower incidence of HCC (Panel **B**). Death was lower in patients with post-SVR HCV but did not differ between patients with non-viral etiologies and those with viremic HCV (Panel **C**). Abbreviations: ALD, Alcohol-associated Liver Disease; HBV, Hepatitis B; HCC, hepatocellular carcinoma; HCV, Hepatitis C; HD, hepatic decompensation; MASLD, Metabolic Dysfunction-Associated Steatotic Liver Disease; SVR, sustained virologic response

The 1- and 2-year cumulative incidence rates of hepatic decompensation per 1000 patients were 150.5 and 173.7 for ALD and 60.3 and 111.2 for MASLD, compared to 58.6 and 90.1 for viremic HCV (Fig. 2A**).**

**Table 2:** Association of liver disease etiology with hepatic decompensation, hepatocellular carcinoma, and death

	Hepatic Decompensation		Hepatocellular Carcinoma		Death	
Covariate	sHR (95% CI)	Incidence Rates 1-yr, 2-yr, 3-year (%)	sHR (95% CI)	Incidence Rates 1-yr, 2-yr, 3-year (%)	sHR (95% CI)	Incidence Rates 1-yr, 2 year, 3-year (%)
Etiology (Reference: Viremic Hepatitis C)	---	5.9, 9.1, 13.7	---	4.8, 7.7, 10.1	---	2.5, 6.0, 10.2
Alcohol-associated liver disease	1.68 (1.10–2.57)	15.1, 17.4, 20.1	0.45 (0.23–0.84)	3.4, 6.3, 6.9	1.14 (0.70–1.85)	3.9, 8.5, 14.5
MASLD	1.52 (0.94–2.45)	6.0, 11.1, 17.5	0.27 (0.12–0.59)	1.1, 1.7, 2.2	1.03 (0.57–1.88)	2.8, 5.0, 11.9
Hepatitis B virus	0.78 (0.18–3.42)	0, 0, 11.2	0.71 (0.16–3.11)	10.5, 10.5, 16.1	1.12 (0.42–2.95)	0, 0, 5.9
Post-SVR Hepatitis C virus	0.86 (0.52–1.41)	5.7, 9.4, 13.8	0.45 (0.24–0.84)	1.4, 2.9, 5.1	0.43 (0.24–0.79)	0.7, 2.2, 4.4
Other	1.34 (0.83–2.16)	2.6, 8.8, 18.8	0.46 (0.22–0.98)	0.8, 1.7, 5.4	0.97 (0.57–1.66)	2.6, 7.9, 15.4

Analyses adjusted for age, sex, race, Child Pugh class, MELD-Na, body mass index, and metabolic comorbid conditions

*Abbreviations*: *MASLD* Metabolic dysfunction-associated steatotic liver disease, *sHR* subdistribution hazard ratio, *SVR* Sustained virologic response

Results were generally consistent in subgroup analyses by type of health system, although none of the associations reached statistical significance. Compared to those with viremic HCV infection, higher hazard of hepatic decompensation was observed for patients with ALD (sHR 1.49; 95% CI 0.95–2.34) or MASLD (sHR 1.41; 95% CI 0.82–2.41) at the safety-net health system as well as those with ALD (sHR 4.78; 95% CI 0.77–29.8) or MASLD (sHR 3.20; 95% CI 0.48–21.6) at the tertiary care referral center.

Results were consistent in subgroup analyses among those with compensated cirrhosis at baseline (*n* = 818) (Table 3). Compared to patients with viremic HCV, those with ALD (sHR 1.89; 95% CI 1.12–3.20) or MASLD (HR 2.24, 95%CI 1.25–4.00) had a higher hepatic decompensation.

**Table 3:** Association of liver disease etiology with hepatic decompensation, hepatocellular carcinoma, and death among those patients with compensated cirrhosis

	Hepatic Decompensation		Hepatocellular Carcinoma		Death	
Covariate	sHR (95% CI)	Incidence Rates 1-yr, 2-yr, 3-year (%)	sHR (95% CI)	Incidence Rates 1-yr, 2-yr, 3-year (%)	sHR (95% CI)	Incidence Rates 1-yr, 2-yr, 3-year (%)
Etiology (Ref: Viremic Hepatitis C)	---	4.8, 7.7, 12.4	---	4.5, 7.4, 9.8	---	2.6, 5.9, 10.3
Alcohol-associated liver disease	1.89 (1.12–3.20)	12.5, 14.3, 18.1	0.48 (0.21–1.11)	3.6, 4.5, 4.5	0.88 (0.46–1.66)	1.8, 5.4, 8.3
MASLD	2.24 (1.25–4.00)	7.1, 12.9, 20.1	0.25 (0.09–0.65)	0, 0.7, 1.5	1.11 (0.52–2.35)	1.4, 2.9, 8.7
Hepatitis B virus	0.90 (0.18–4.45)	0, 0, 14.3	0.91 (0.21–4.01)	7.1, 7.1, 14.3	0.99 (0.29–3.36)	0, 0, 7.1
Post-SVR Hepatitis C virus	0.76 (0.43–1.36)	5.7, 9.8, 10.6	0.47 (0.24–0.92)	1.6, 3.3, 5.7	0.38 (0.19–0.76)	0.8, 1.6, 3.3
Other	1.51 (0.87–2.62)	1.1, 5.6, 14.7	0.38 (0.15–0.96)	0, 1.1, 4.6	1.46 (0.78–2.73)	1.1, 6.7, 15.9

Analyses adjusted for age, sex, race, Child Pugh class, MELD-Na, body mass index, and metabolic comorbid conditions

*Abbreviations*: *MASLD* Metabolic dysfunction-associated steatotic liver disease, *sHR* Subdistribution hazard ratio, *SVR* sustained virologic response

### Factors associated with HCC

In multivariable analyses adjusted for demographics, liver dysfunction, and metabolic syndrome comorbidities, incident HCC was independently associated with liver disease etiology (Table 2). Compared to patients with viremic HCV, those with MASLD (sHR 0.27; 95% CI 0.12–0.59), ALD (sHR 0.45; 95% CI 0.23–0.84), post-SVR HCV (sHR 0.45; 95% CI 0.24–0.84), or other non-viral etiologies (sHR 0.46; 95% CI 0.22–0.98) had a significantly lower hazard of HCC. Results were generally consistent in subgroup analyses by type of health system. At the safety-net health system, patients with MASLD (sHR 0.35; 95% CI 0.14–0.85) and post-SVR HCV (sHR 0.37; 95% CI 0.17–0.81) had lower hazard of HCC compared to those with viremic HCV infection. Patients with ALD also had lower hazards although this did not reach statistical significance (sHR 0.56; 95% CI 0.28–1.12). At the tertiary care referral center, patients with MASLD (sHR 0.04; 95% CI 0.00–0.44) and ALD (sHR 0.08; 95% CI 0.01–0.82) had lower hazard of HCC compared to those with viremic HCV infection. Patients with post-SVR HCV infection also had lower hazards although this did not reach statistical significance (sHR 0.27; 95% CI 0.05–1.63).

The cumulative 1- and 2-year incidences of HCC per 1000 person-years were 33.7 and 62.7 for ALD, 10.9 and 16.6 for MASLD, and 14.3 and 28.8 for post-SVR HCV, compared to 47.7 and 76.7 for viremic HCV (Fig. 2B).

Results were consistent in subgroup analyses among patients with compensated cirrhosis (*n* = 818) (Table 3). Compared to patients with viremic HCV, those with MASLD (sHR 0.25; 95% CI 0.09–0.65), ALD (sHR 0.48; 95% CI 0.21–1.11), post-SVR HCV (sHR 0.47; 95% CI 0.24–0.92), and other non-viral etiologies (sHR 0.38; 95% CI 0.15–0.96) had a lower hazard of HCC.

### Factors associated with death

In multivariable analyses adjusted for demographics, liver dysfunction, and metabolic syndrome comorbidities, death was associated with liver disease etiology (Table 2). Compared to patients with viremic HCV, those with post-SVR HCV had a lower risk of death (sHR 0.43; 95% CI 0.24–0.79). The risk of death did not significantly differ between patients with MASLD or ALD and those with viremic HCV (Fig. 2C). The cumulative 1- and 2-year incidences of death per 1000 person-years were 7.2 and 21.7 for post-SVR HCV, compared to 25.1 and 60.2 for viremic HCV, 39.0 and 85.2 for ALD, and 27.5 and 49.8 for MASLD (Fig. 2C). Results were consistent in patients with compensated cirrhosis at baseline, with lower hazards of death among patients with post-SVR HCV (sHR 0.38; 95% CI 0.19–0.76) than those with viremic HCV. (Table 3).

## Discussion

In our cohort of over 1000 patients with cirrhosis, we found that liver disease etiology was independently associated with the risk of incident hepatic decompensation and HCC. Specifically, patients with non-viral etiologies of liver disease, including MASLD and ALD, had a significantly higher incidence of hepatic decompensation and lower incidence of HCC. Conversely, death was lower in patients with post-SVR HCV but did not differ between patients with non-viral etiologies and those with viremic HCV. These data suggest that the burden of decompensated cirrhosis in the United States, as well as globally, may markedly increase with the changing epidemiology of chronic liver disease.

The higher incidence of hepatic decompensation in patients with non-viral etiologies may be due to differential susceptibility to ascites and hepatic encephalopathy. Specifically, patients with MASLD appear to decompensate at lower hepatic venous pressure gradient (HVPG) levels than those with viral disease [29]. This finding may be due to increased intrahepatic resistance at the presinusoidal level, such that metabolic derangements damage the liver endothelium differently than in viral disease. These data underscore the importance of interventions to reduce the risk of hepatic decompensation in these patients. Weight loss and glycemic control may reduce the risk of hepatic decompensation in patients with MASLD [30–32]. These interventions enhance metabolic homeostasis, reduce intrahepatic lipid content, and mitigate fibrosis progression, thereby reducing the risk of hepatic decompensation. Pharmacologic therapies for MASLD also likely impact natural history including risk of decompensation. Glucagon-like peptide-1 (GLP-1) receptor agonists, dual gastric inhibitory polypeptide (GIP)/GLP-1 receptor agonists, and pioglitazone have demonstrated efficacy in decreasing hepatic steatosis, resolving MASH, and reducing fibrosis in patients with type 2 diabetes and MASLD or MASH. In the MAESTRO-NASH clinical trial, treatment with resmetirom led to resolution of steatohepatitis without worsening of fibrosis and improvement in fibrosis without worsening of steatohepatitis, leading to FDA approval [33]. With numerous ongoing clinical trials on fibroblast growth factor 21 (FGF21) analogs, sodium-glucose cotransporter-2 (SGLT2) inhibitors, GLP-1 receptor agonists, lipogenesis inhibitors, peroxisome proliferator-activated receptor (PPAR) agonists, thyromimetics, and drug combinations, there will likely be more pharmaceutical therapies to come for MASLD [34, 35].

Our finding of increased risk of hepatic decompensation in ALD could be explained by the direct hepatotoxic effects of alcohol, including oxidative stress, impaired hepatocyte metabolism, and increased gut permeability leading to endotoxemia and inflammation [36]. Over time, this leads to an accumulation of fat in the liver, and the subsequent inflammation and fibrosis predispose to decompensation. Patients with alcohol-related cirrhosis who maintain abstinence have a markedly lower risk of decompensation compared to those with active drinking [37]. Abstinence reduces the cumulative incidence of decompensation in patients with clinically significant portal hypertension as well as those with severe portal hypertension [37, 38]. In a subset of patients with decompensated alcohol-related cirrhosis, abstinence can even lead to hepatic recompensation with resolution of ascites and hepatic encephalopathy, absence of variceal bleeding, and improvement in liver function [39].

Our results are consistent with multiple studies that have shown that the risk of HCC is generally lower in non-viral etiologies of cirrhosis. Kanwal et al. found that the annual incidence rate of HCC was 1.7% in patients with post-SVR HCV, 1.3% in patients with ALD, and 1.2% in patients with MASLD cirrhosis [5]. Similarly, in a study by Ioannou et al., patients with HCV-related cirrhosis had more than three times the incidence of HCC (3.3 per 100 patient-years) compared to those with ALD (0.86 per 100 patient-years) or MASLD (0.90 per 100 patient-years) [40]. There is also reported lower adherence to HCC surveillance among patients with alcohol-associated or MASH-related cirrhosis, which may explain the lower incidence of HCC in these populations due to a lack of detection through screening [41–44]. Although we found a lower risk of HCC in non-viral etiologies, the number of at-risk individuals is expected to rise with growing incidences of metabolic syndrome, obesity, type 2 diabetes, MASLD, as well as increasing alcohol consumption. This increased denominator of at-risk individuals may overwhelm the reduction in individual risk, such that the overall burden of HCC may increase over time. Recent epidemiologic data demonstrate lower HCC incidence and mortality, but it is unclear if this will continue to decrease over time [45].

Aside from liver disease etiology, we observed other well-reported risk factors including older age for risk of HCC and sex for hepatic decompensation [46, 47]. We did not find significant differences in hepatic decompensation or HCC among non-Hispanic Black or Hispanic patients compared to non-Hispanic Whites, which is discordant with other studies [48]. Rich et al. reported a disproportionate burden of disease in American Indian, Hispanic, and Black patients compared to non-Hispanic White patients [49]. Furthermore, a study on U.S. veterans with cirrhosis found that compared with non-Hispanic White patients, Hispanic patients had a higher risk for HCC overall, particularly those with ALD and MASLD, whereas non-Hispanic Black patients had a lower risk for HCC [50]. The same study also found a lower risk for hepatic decompensation overall in Black patients across all etiologies of cirrhosis.

We acknowledge several limitations of our study. Its retrospective study design is prone to measurement bias, residual confounding, as well as possible missed ascertainment of outcomes for patients lost to follow-up. Notably, alcohol use itself is often underreported, which could result in misclassification of liver disease etiology. Second, we captured risk factors for hepatic decompensation and HCC at the time of patients’ index visit but did not use time-varying covariates to account for subsequent events such as receipt of HCV treatment. Third, we focused on incident ascites, hepatic encephalopathy, and HCC, but we did not examine other cirrhosis complications including variceal bleeding and hepatorenal syndrome as these acute decompensation events can often occur at outside institutions and are prone to ascertainment bias. Fourth, we did not capture factors that may influence natural history including the severity of obesity and diabetes, as well as alcohol use patterns. We believe these limitations are offset by strengths including the large size of our cohort, its follow-up period of several years, and its diversity in terms of race and liver disease etiologies.

In conclusion, the etiology of liver disease is significantly associated with risk of hepatic decompensation and HCC in patients with cirrhosis, with non-viral etiologies being associated with higher risk of hepatic decompensation and lower risk of HCC. Therefore, the changing epidemiology of liver disease will likely have a profound impact on the burden of these cirrhosis complications.

## Supplementary Information

Supplementary Material 1.

## Data Availability

The data supporting the conclusions of this article are included within the article, including tables and figures. Additional data are available from the corresponding author upon reasonable request.
