# Supplementary material for: Etiology of cirrhosis is associated with risk of hepatic decompensation and hepatocellular carcinoma
Source: BMC Gastroenterol. 2025 Dec 9;26:22. doi: 10.1186/s12876-025-04538-y (PMC12802128; doi:10.1186/s12876-025-04538-y)
Supplement: Supplementary file 1 — Supplementary Material 1. [file 12876_2025_4538_MOESM1_ESM.docx]

**Supplemental Table 1. ICD-9 codes for identification of patients with cirrhosis**

| **ICD-9 Code** | **Meaning** |
| --- | --- |
| 456.0 | Esophageal varices with bleeding |
| 456.1 | Esophageal varices without bleeding |
| 456.2 | Varices in diseases classified elsewhere with bleeding |
| 456.21 | Varices in diseases classified elsewhere without bleeding |
| 567.23 | Spontaneous bacterial peritonitis |
| 571.2 | Alcohol cirrhosis |
| 571.5 | Cirrhosis without alcohol |
| 572.2 | Hepatic encephalopathy |
| 572.3 | Portal hypertension |
| 572.4 | Hepatorenal syndrome |
| 789.59 | Ascites |
